# Labeling Ebola Virus with a Self-Splicing Fluorescent Reporter

**DOI:** 10.3390/microorganisms10112110

**Published:** 2022-10-26

**Authors:** Baylee Heiden, Elke Mühlberger, Christopher W. Lennon, Adam J. Hume

**Affiliations:** 1 Department of Microbiology, Boston University School of Medicine, Boston, MA 02118, USA; 2 National Emerging Infectious Diseases Laboratories, Boston University, Boston, MA 02118, USA; 3 Department of Biological Sciences, Murray State University, Murray, KY 42071, USA; 4 Center for Emerging Infectious Diseases Policy & Research, Boston University, Boston, MA 02118, USA

**Keywords:** intein, Ebola virus, reporter gene

## Abstract

Inteins (intervening proteins) are polypeptides that interrupt the sequence of other proteins and remove themselves through protein splicing. In this intein-catalyzed reaction, the two peptide bonds surrounding the intein are rearranged to release the intein from the flanking protein sequences, termed N- and C-exteins, which are concurrently joined by a peptide bond. Because of this unique functionality, inteins have proven exceptionally useful in protein engineering. Previous work has demonstrated that heterologous proteins can be inserted within an intein, with both the intein and inserted protein retaining function, allowing for intein-containing genes to coexpress additional coding sequences. Here, we show that a fluorescent protein (ZsGreen) can be inserted within the *Pyrococcus horikoshii* RadA intein, with the hybrid protein (ZsG-Int) maintaining fluorescence and splicing capability. We used this system to create a recombinant Ebola virus expressing a fluorescent protein. We first tested multiple potential insertion sites for ZsG-Int within individual Ebola virus proteins, identifying a site within the VP30 gene that facilitated efficient intein splicing in mammalian cells while also preserving VP30 function. Next, we successfully rescued a virus containing the ZsG-Int-VP30 fusion protein, which displayed fluorescence in the infected cells. We thus report a new intein-based application for adding reporters to systems without the need to add additional genes. Further, this work highlights a novel reporter design, whereby the reporter is only made if the protein of interest is translated and does not remain fused to the protein of interest.

## 1. Introduction

Intervening proteins (inteins) are abundant in the genomes of archaea and bacteria, but are also present in unicellular eukaryotes, phages, and viruses [1]. Inteins are enzymatic protein sequences that interrupt the sequence of other proteins. Inteins are synthesized within other proteins after which they are removed through a self-catalyzed protein splicing reaction [2]. In this process, the intein excises itself from the flanking protein sequences, termed N- and C-exteins, rejoining the exteins seamlessly with a peptide bond [2].

Inteins come in diverse configurations. Full-length inteins house homing endonuclease domains (HEN) between conserved sequences that facilitate intein catalysis, known as splicing blocks [2]. The HEN is used for horizontal mobility at the DNA level, conferring the ability to invade intein-minus alleles. Mini-inteins, which do not contain an HEN domain, retain the splicing blocks needed for self-excision and have a loop in place of the HEN [2]. Most inteins found in nature are either full-length or mini, with the intein and the interrupted protein translated as a single polypeptide that undergoes cis splicing. 

Inteins are self-contained, single-turnover enzymes with a unique ability to rearrange peptide bonds to remove the intein while simultaneously ligating flanking exteins. This ability has been exploited in numerous protein engineering applications including bioseparations, bioconjugation, biosensing, and protein cyclization [3]. Previous work has demonstrated that exogenous proteins can be inserted within inteins in place of the HEN, are correctly spliced, and maintain function [4,5,6]. In one example, an intein housing a selectable marker was employed to internally label proteins with GFP that remains part of the final product [6].

Ebola virus (EBOV) is a nonsegmented negative sense RNA virus that belongs to the filovirus family. With case fatality rates ranging from 40 to 90%, EBOV is the most pathogenic filovirus species [7]. One intriguing application for inteins is their potential use to make reporter viruses. Currently, generating reporter viruses involves one of three main approaches: (1) adding reporter genes as additional transcriptional units, (2) making polyprotein fusions of reporter proteins and viral proteins, or (3) usage of the 2A ribosomal skipping mechanism. Incorporating reporter genes as additional transcriptional units or by fusing them to viral proteins can result in a fitness cost and may limit their suitability in certain assays [8,9,10,11,12], and the 2A system in some circumstances can result in hindered protein functions [13,14,15]. Here, we describe an intein-based self-removing fluorescent reporter for labeling EBOV. Our reporter design provides a proof-of-principle for a novel methodology that avoids some of these limitations and represents another tool for molecular virologists.

## 2. Materials and Methods

### 2.1. Biosafety Statement

All work with wildtype and recombinant Ebola virus (EBOV) was performed in the biosafety level 4 (BSL-4) facility of Boston University’s National Emerging Infectious Diseases Laboratories (NEIDL) following the approved standard operating procedures in compliance with local and national regulations pertaining to handling BSL-4 pathogens and Select Agents.

### 2.2. Bacterial Expression Plasmid Cloning

The pMAL-ZsG-Int-His plasmid DNA was synthesized, subcloned, and sequenced by Genscript. The ZsG-Int protein sequence can be found in File S1. 

### 2.3. MBP-ZsG-Int-His Expression in Escherichia coli

*E. coli* NEB5a containing pMAL-ZsG-Int-His was grown to an OD_600_ of 0.4–0.6 at 37 °C in LB containing 100 mg/mL ampicillin (for plasmid maintenance) and protein expression was induced by the addition of Isopropyl β-D-1-thiogalactopyranoside to a final concentration of 1 mM. Protein was expressed for 2 h at 37 °C and pelleted by centrifugation. Fluorescent cell pellets were visualized using an Amersham Imager 680 (GE Healthcare, Chicago, IL, USA).

### 2.4. Isolation of His-Tagged Proteins Expressed in E. coli and SDS-PAGE

Following protein expression, cells were resuspended in Buffer A (20 mM Tris-HCl, 500 mM NaCl, 30 mM Imidazole, pH 8.0) and lysed by sonication. Insoluble material was removed by centrifugation and the soluble lysate was mixed with Ni-charged MagBeads (Genscript, Piscataway, NJ, USA) preequilibrated in Buffer A, washed with Buffer A, and eluted with Buffer B (20 mM Tris-HCl, 500 mM NaCl, 300 mM Imidazole, pH 8.0). Samples were mixed with 4X Bolt LDS Sample Buffer (Invitrogen, Waltham, MA, USA) containing 4% β-mercaptoethanol, heated to 95 °C for 5 min, separated using 8–16% Sure-PAGE Bis-Tris gels (Genscript) with MOPS running buffer, stained by Coomassie, and visualized using an Amersham Imager 680 (GE Healthcare).

### 2.5. Eukaryotic Cell Lines

Eukaryotic cell lines used in this study include human embryonic kidney cells (293T; ATCC CRL-3216, African green monkey kidney cells (Vero E6; ATCC CRL-1586), human liver cells (Huh7; kindly provided by Apath L.L.C., New York, NY, USA), and golden hamster baby kidney cells (BHK-21; ATCC CCL-10). 293T, Vero E6, BHK-21, and Huh7 cells were maintained in Dulbecco’s modified Eagle medium (DMEM) supplemented with 10% fetal bovine serum (FBS), L-glutamine (200 mM), and either penicillin (50 units/mL) and streptomycin (50 mg/mL) or 100 ug/mL Primocin. All cell lines were grown at 37 °C/5% CO_2_.

### 2.6. Viral Sequences

The Zaire ebolavirus isolates, Ebola virus/H.sapiens-tc/COD/1976/Yambuku-Mayinga (NC_002549) NCBI reference filovirus sequence was used for cloning.

### 2.7. Eukaryotic Expression Plasmids

EBOV support plasmids (pCAGGS-NP_EBOV_, -VP35_EBOV_, -VP30_EBOV_, and -L_EBOV_) were described previously [16]. A plasmid expressing a codon-optimized version of T7 RNA polymerase was previously described [17]. A pTwist-Amp plasmid containing the ZsG-Int1 insert for VP35 was ordered from Twist Bioscience. The sequence can be found in File S1. pCAGGS-VP35_EBOV_ was digested with KpnI, AgeI, and DpnI and the Genscript vector was digested with ZraI and ScaI-HF (NEB). Fragments were gel purified and cloned using NEBuilder HiFi DNA Assembly (NEB). 

A pTwist-Amp plasmid containing the ZsG-Int2 insert for VP35 was ordered from Twist Bioscience. The sequence can be found in File S1. pCAGGS-VP35_EBOV_ was digested with AgeI, NheI, and DpnI and the Genscript vector was digested with DraI and PsiI-v2 (NEB). Fragments were gel purified and cloned using NEBuilder HiFi DNA Assembly (NEB).

A pTwist-Amp plasmid containing the ZsG-Int insert for VP30 (pTwist-Amp-ZsG-Int) was ordered from Twist Bioscience. The sequence can be found in File S1. pCAGGS-VP30_EBOV_ was digested with Bsu36I, BstZ171-HF, and DpnI and the Genscript vector was digested with PvuII-HF and EcorRV (NEB). Fragments were gel purified and cloned using NEBuilder HiFi DNA Assembly (NEB).

A DNA fragment containing the ZsG-Int-AA insert for VP30 was ordered from Twist Bioscience. The sequence can be found in File S1. pCAGGS-VP30_EBOV_ was digested with PvuII-HG, EcoRV, and DpnI and gel purified. Fragments were cloned using NEBuilder HiFi DNA Assembly (NEB).

### 2.8. Cloning of EBOV Minigenomes

To generate the p2,0-3E5E-mCherry plasmid, mCherry was first amplified from a pCAGGS-mCherry plasmid using primers GTCCATATGATGGTGAGCAAGGGCGAG-GAGGATAAC and GATGCGGCCGCCTACTTGTACAGCTCGTCCATGCC. The mCherry PCR product was then inserted into the p2,0-3E5E-CAT plasmid [18] via NdeI and Not I restriction digest and ligation. 

To generate the p2,0-3E5E-NanoLuc plasmid, NanoLuc was first amplified from a pCS2-NanoLuc plasmid using primers caaaattccgagtatgtctcatatgATGGTCTTCACACTCGAAGATTTCG and gcaagtatcaggatactatgcggccgcCGCCAGAATGCGTTCGC. The NanoLuc PCR product was then inserted into the p2,0-3E5E-GFP plasmid [11] and digested with NdeI and NotI (NEB) using NEBuilder HiFi DNA Assembly (NEB). 

### 2.9. Cloning of Full-Length EBOV Plasmid

The full-length EBOV plasmid p15AK-EBOV was described previously [19]. Four fragments were generated in order to clone p15AK-EBOV-VP30-ZsG-Int. The first fragment was generated by digesting p15AK-EBOV with ApaI and SacI (NEB), treating with rSAP (NEB), and gel purifying a 15.1 kb fragment. The second fragment was generated by PCR amplifying a 4.4 kb fragment from p15AK-EBOV using primers TCCTTGAAGCTATGGTGAATG and TGTGTCTCACATAAAAGACTCAGC, followed by DpnI digestion and column purification. The third fragment was generated by digesting pTwist-Amp-ZsG-Int with PvuII and EcoRV (NEB), and gel purifying a 1.6 kb fragment. The fourth fragment was generated by PCR amplifying a 1.6 kb fragment from p15AK-EBOV using primers CACAATACTCCGTATACCTATCATCA and GTTCATTGCCGATTTGTCG, followed by DpnI digestion and column purification. p15AK-EBOV-VP30-ZsG-Int was then cloned by assembling these four fragments using NEBuilder HiFi DNA Assembly (NEB). 

### 2.10. Individual Plasmid Transfections

Individual expression plasmids were transfected as follows. Briefly, 1 × 10^5^ 293T cells were seeded in a 12-well plate one day prior to transfection. The next day, cells were transfected with individual expression plasmids for VP35, VP35-ZsG-Int1, VP35-ZsG-Int2, VP30, VP30-ZsG-Int, and VP30-ZsG-Int-AA using TransIT-LT1 per manufacturer’s recommendations (Mirus Bio LLC, Madison, WI, USA).

### 2.11. EBOV Minigenome Transfections

EBOV minigenome transfections were performed as described previously [20]. Briefly, 1 × 10^5^ Huh7 or BHK21 cells were seeded in a 12-well plate one day prior to transfection. The next day, cells were transfected with EBOV minigenome plasmid DNA (containing either a NanoLuc or mCherry reporter) along with EBOV support plasmids encoding NP, VP35, VP30, and L as well as a plasmid expressing codon-optimized T7 RNA polymerase. Expression plasmids for wild-type VP35 and VP30 were substituted with expression plasmids for ZsG-Int-containing versions (VP35-ZsG-Int1 or VP35-ZsG-Int2 for VP35, VP30-ZsG-Int or VP30-ZsG-Int-AA for VP30) as indicated. As negative controls for EBOV minigenome transfections, empty pCAGGS plasmid was used instead of L plasmid. Transfection of Huh7 or BHK21 cells was performed using TransIT-LT1 per the manufacturer’s recommendations (Mirus Bio LLC).

### 2.12. Recombinant EBOV Rescue Transfection

Recombinant EBOV rescue transfection was performed as described previously [17]. Briefly, 1:1 mixtures of Huh7:Vero E6 cells (1 × 10^5^ cells plated per well of a 12-well plate) were transfected with EBOV support plasmids (125 ng pCAGGS-NP_EBOV_, 125 ng pCAGGS-VP35_EBOV_, 50 ng pCAGGS-VP30_EBOV_, and 500 ng pCAGGS-L_EBOV_), pCAGGS-T7 (20 ng; codon-optimized), and the recombinant EBOV-VP30-ZsG-Int full-length plasmid (1 μg) using TransIT-LT1 per manufacturer’s recommendations (Mirus Bio LLC). Media was changed approximately 18 h post-transfection and cells were monitored for cytopathic effect (CPE) and fluorescence for ZsGreen. Supernatants of cells showing CPE and fluorescence were transferred to T75 flasks of Vero E6 cells approximately 7–11 days post-transfection. Rescue of recombinant EBOV was performed in the BSL-4 facility of the NEIDL, following BSL-4 biosafety procedures.

### 2.13. Virus Propagation

EBOV Mayinga virus isolate was kindly provided by the NIH NIAID Rocky Mountain laboratories. Virus stocks were grown in Vero E6 cells, as previously described [21]. Virus titers were determined in Vero E6 cells by 50% tissue culture infectious dose (TCID_50_) assay and calculated using the Spearman-Kärber algorithm. All work with EBOV was performed under BSL-4 conditions at the NEIDL, following approved SOPs.

### 2.14. Growth Curve Infections

Growth curve infections were performed as described previously [17]. Briefly, Huh7 cells (7 × 10^4^ cells plater per well of a 24-well plate) were infected with the indicated viruses at an initial MOI of 0.1. At the indicated times post-infection supernatant was clarified and virus titers were determined in Huh7 cells by TCID_50_ assay and calculated using the Spearman-Kärber algorithm.

### 2.15. Minigenome NanoLuc LUCIFERASE Assays

At 3 days post-transfection, cell lysates were harvested in Nano-Glo Luciferase Assay Buffer (Promega, Madison, WI, USA) and analyzed with the Nano-Glo Luciferase Assay System (Promega) using a LUMIstar Omega luminometer (BMG LabTech, Ortenberg, Germany). Cell lysates were diluted in nuclease-free H_2_O as needed. Luciferase values were then calculated as a fold increase over the negative control (minus L) values. The standard error of the mean (SEM) and two-tailed t-tests for these data was calculated using GraphPad Prism software.

### 2.16. Live-Cell Fluorescence Microscopy

Cells transfected with individual expression plasmids or transfected with EBOV minigenomes containing a mCherry reporter, as described above, were imaged by live-cell fluorescence microscopy and images were taken using a Nikon TS100 Eclipse microscope and Nikon DS Qi1Mc camera with NIS Elements F software.

### 2.17. Immunofluorescence Analysis

Immunofluorescence to detect EBOV was performed as described previously [20]. Briefly, a total of 1 × 10^5^ Huh7 cells were seeded per well of an 8-well chamber slide (Nunc Lab-Tek II) and 1 day later infected with EBOV or rEBOV-VP30-ZsG-Int at a multiplicity of infection (MOI) of 3 TCID_50_ units per cell. At the indicated time points post-infection, cells were inactivated and fixed by treatment with 10% formalin for 6 h. Cells were washed 4 times with phosphate-buffered saline (PBS), permeabilized with a mixture of acetone and methanol (1:1, *v*/*v*) for 5 min at −20 °C, washed 4 times with PBS, treated with 0.1 M glycine for 5 min at room temperature, washed 4 times with PBS, and then incubated in blocking solution (2% bovine serum albumin, 0.2% Tween 20, 3% glycerol, and 0.05% sodium azide in PBS) for 1 h at room temperature. The primary antibody used for the detection of EBOV VP30 (rabbit α-VP30 diluted 1:100 in blocking solution, kind gift from V. Volchkov, Claude Bernard University of Lyon, Lyon, France) and EBOV NP (rabbit α-NP diluted 1:200 in blocking solution, IBT, 0301-012) were added and incubated overnight at 4 °C. Cells were washed 4 times with PBS and then incubated with secondary antibody (AlexaFluor 594 diluted 1:100 in blocking solution, Invitrogen) in the presence of DAPI. Immunofluorescence analysis was performed three times in independent experiments. Slides were then mounted and imaged using a Nikon Ti2 Eclipse microscope and Photometrics Prime BSI camera with NIS Elements AR software.

### 2.18. Western Blots

Western blots were performed as described previously [22]. The following primary antibodies were used: mouse α-ZsGreen (Origene, TA180002), mouse α-tubulin (Sigma, T6199), rabbit α-VP30 (kind gift from M. Javad Aman, Integrated Biotherapeutics, Rockville, MD, USA), goat α-VP35 (Antagene, custom), rabbit α-NP (IBT, 0301-012). The following secondary antibodies were used: donkey α-rabbit 680 (LiCor, 926-68073), donkey α-mouse 800 (LiCor, 926-32212), and donkey α-goat 800 (LiCor, 926-32214). Western blots were visualized and quantified with the LI-COR Odyssey imaging system. The standard error of the mean (SEM) and two-tailed t-tests for these data was calculated using GraphPad Prism software.

## 3. Results

### 3.1. Splicing of an Intein-Based Reporter in E. coli

**Figure 1:**
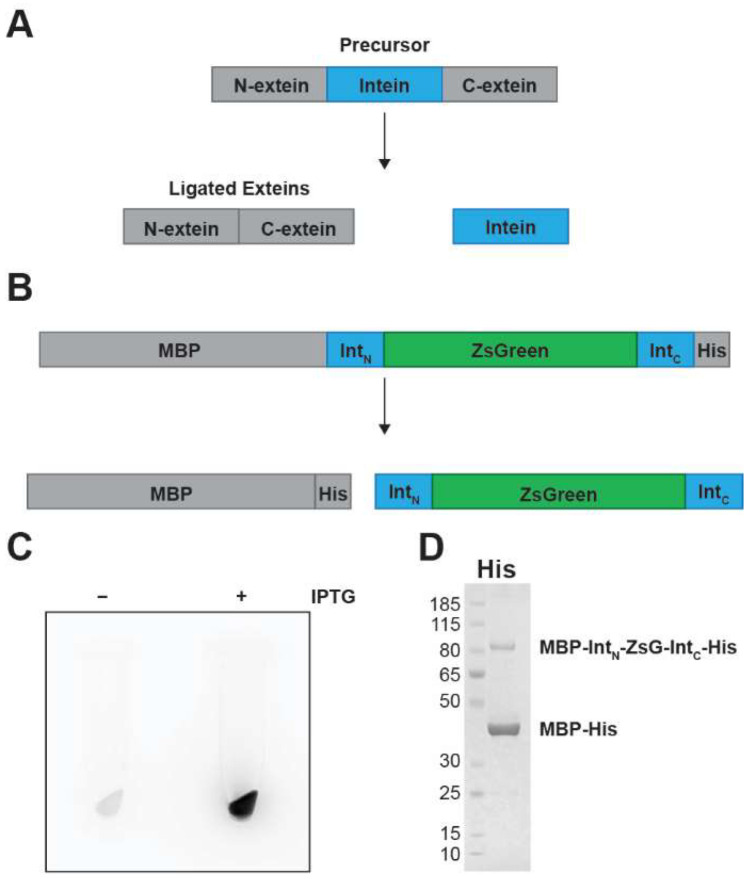
Protein splicing and ZsG-Int fusion splicing in *E. coli*. (**A**) Diagram of intein-containing precursor protein. Following protein splicing, the two peptide bonds flanking the intein are rearranged into one, linking the N- and C-exteins together (ligated exteins; gray) and releasing the intein (blue). (**B**) Diagram of intein-housing ZsGreen construct (ZsG-Int) for expression in *E. coli.* Maltose-binding protein (MBP) and a His-tag (His) serve as the exteins. Length is relative to the number of residues. ZsGreen is inserted within the intein, with the upstream (IntN) and downstream (IntC) regions of the intein in blue, and ZsGreen in green. (**C**) *E. coli* cell pellets containing the MBP-IntN-ZsGreen-IntC-His expression plasmid under 460 nm light. In the absence of the inducer IPTG, cells display minimal fluorescence. However, when grown in the presence of IPTG, cells display strong fluorescence, indicating ZsGreen expression and folding (**D**) ZsG-Int is splicing active following expression in *E. coli*. MBP-IntN-ZsGreen-IntC-H was expressed and isolated using Ni2+-affinity resin. Eluate from Ni2+-affinity resin was separated by SDS-PAGE and stained by Coomassie Brilliant Blue. Major product is consistent in size with the expected ligated exteins, MBP-His (43.2 kDa). Minor product is consistent in size with the expected unspliced precursor, MBP-IntN-ZsGreen-IntC-His (89.4 kDa).

We reasoned that a fluorescent protein could be inserted into an intein to yield a self-excising fluorescent reporter (Figure 1A,B). Compared to other translational reporter designs where the reporter is fused to the protein of interest, a fluorescent protein housed within an intein remains within the excised intein product following splicing. To test this design, we inserted ZsGreen [23] (ZsG) within the RadA intein from the archaeon *Pyrococcus horikoshii* (Pho). Pho RadA is a mini-intein that naturally lacks a HEN and was identified as being exceptionally efficient at splicing within foreign exteins [24]. ZsGreen, flanked by short glycine-serine linkers, was inserted between residues 126 and 127 of the Pho RadA intein within an unstructured loop that presumably once housed a HEN [25].

We first examined our ZsG-containing intein (ZsG-Int) for the ability to both produce a fluorescent signal and splice efficiently in Escherichia coli when using the maltose-binding protein (MBP) as the N-extein and a His-tag as the C-extein (Figure 1B). Upon induction with IPTG, a fluorescent signal was observed in the cells (Figure 1C). To measure splicing efficiency, we isolated His-tagged proteins by pull-down with magnetic Ni-NTA resin. We determined splicing efficiency by comparing the amounts of precursor (MBP-ZsG-Int-His) and ligated exteins (MBP-His). We found that the overwhelming majority of product retained by the resin was consistent with the size of MBP-His (Figure 1D), indicating that ZsG-Int splices efficiently.

### 3.2. Insertion and Splicing of an Intein-Based Reporter in Ebola Virus Proteins

To explore if inteins can be used to introduce reporter genes into viral genomes, we chose the Ebola virus (EBOV) as our exemplar. EBOV causes severe disease in humans and is classified as a biosafety level 4 agent [7]. The negative-sense RNA genome of EBOV contains seven genes [26]. Recombinant Ebola viruses expressing a fluorescent reporter gene have been widely used as valuable tools in tropism studies, live-cell imaging, inactivation studies, and antiviral drug screening assays [17,27,28,29]. Incorporating a reporter gene into recombinant EBOV currently has been achieved with a variety of methods including introducing additional transcriptional units, expressing reporters as fusion proteins with viral proteins, and using a 2A peptide system [17,27,30]. Drawbacks to these systems exist; however, additional transcriptional units can interfere with ratios of viral mRNAs and proteins, fusion proteins can reduce the functionality of viral proteins, and the 2A system incorporates potentially undesired RNA secondary structures into viral genomes and leaves either a residual N-terminal proline or C-terminal 2A peptide on the viral protein [13]. Incorporation of modified inteins within viral open reading frames represents a method by which reporter genes could potentially be added to viruses while avoiding these drawbacks.

**Figure 2:**
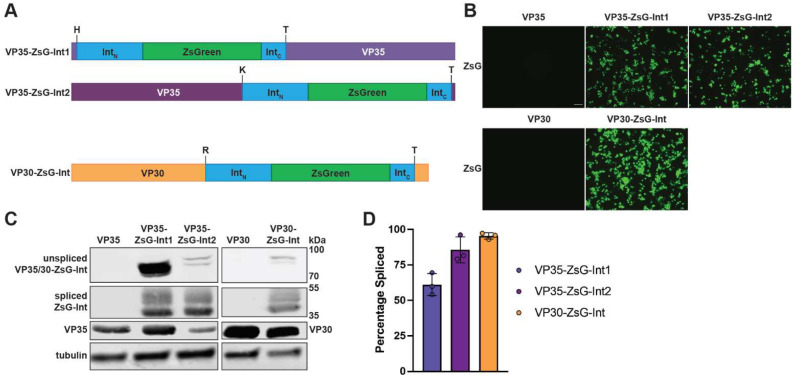
Expression and splicing efficiency of two Ebola virus (EBOV) proteins containing ZsG-Int. (**A**) Diagram of EBOV VP35 and VP30 fusion proteins used in this study. Length is relative to the number of residues and insertion position. Amino acid residues flanking the intein insertion are shown above each diagram. VP35 in purple, VP30 in orange, and remaining coloring as in Figure 1. (**B**) Expression of functional ZsGreen in 293T cells expressing wt and ZsG-Int-containing VP35 and VP30 constructs at 2 days post-transfection (dpt). Scale bar = 100 µm. Images were taken with 10× objective. (**C**) Western blots of 293T cells expressing wt and ZsG-Int-containing VP35 and VP30 constructs at 2 dpt. Detection of unspliced proteins VP35-ZsG-Int1/2 (83.6 kDa) and VP30-ZsG-Int (78.8 kDa) as well as spliced products ZsG-Int (46.2 kDa), VP35 (37.4 kDa), and VP30 (32.6 kDa). (**D**) Quantification of intein splicing efficiency shown in C based on 3 replicates. Quantification of bands performed in Image Studio. Splicing efficiency is calculated by dividing the amount of spliced intein (ZsG-Int) by total intein expression products (spliced + unspliced). Experiments have been performed 3 times with similar outcomes with representative results shown.

We first sought to determine a suitable insertion site for the modified RadA intein containing ZsGreen within the EBOV genome. Individual EBOV proteins were searched to identify potential sites for intein insertion using multiple criteria. Firstly, we considered the requirement of a nucleophilic residue immediately following the intein (+1 position), which for the Pho RadA intein is a threonine. The residue preceding the intein (−1 position) also influences splicing efficiency [25]. We, therefore, limited our search for insertion sites where the −1 position amino acid would yield > 90% splicing efficiency based on prior testing (M, K, H, N, R, S, or L) followed immediately by a threonine [25]. Secondly, we sought to insert the intein sequence near the N- or C-terminus of viral ORFs to reduce any potential impact on viral protein folding. We identified 3 locations to test for intein insertion within EBOV proteins: two within the viral polymerase cofactor VP35 [26] and one within the viral transcription factor VP30 [26] (Figure 2A) and made expression constructs for each of these modified intein-containing genes (VP35-ZsG-Int1, VP35-ZsG-Int2, and VP30-ZsG-Int). These ZsG-Int-containing proteins were each efficiently expressed in mammalian cells as determined by fluorescence microscopy and Western blotting (Figure 2B,C). Similar to what was observed in *E. coli*, the splicing efficiency of ZsG-Int appeared high, particularly for VP30-ZsG-Int (Figure 2C,D and Figure S1).

### 3.3. Functionality of Ebola Virus Proteins Containing an Intein-Based Reporter

**Figure 3:**
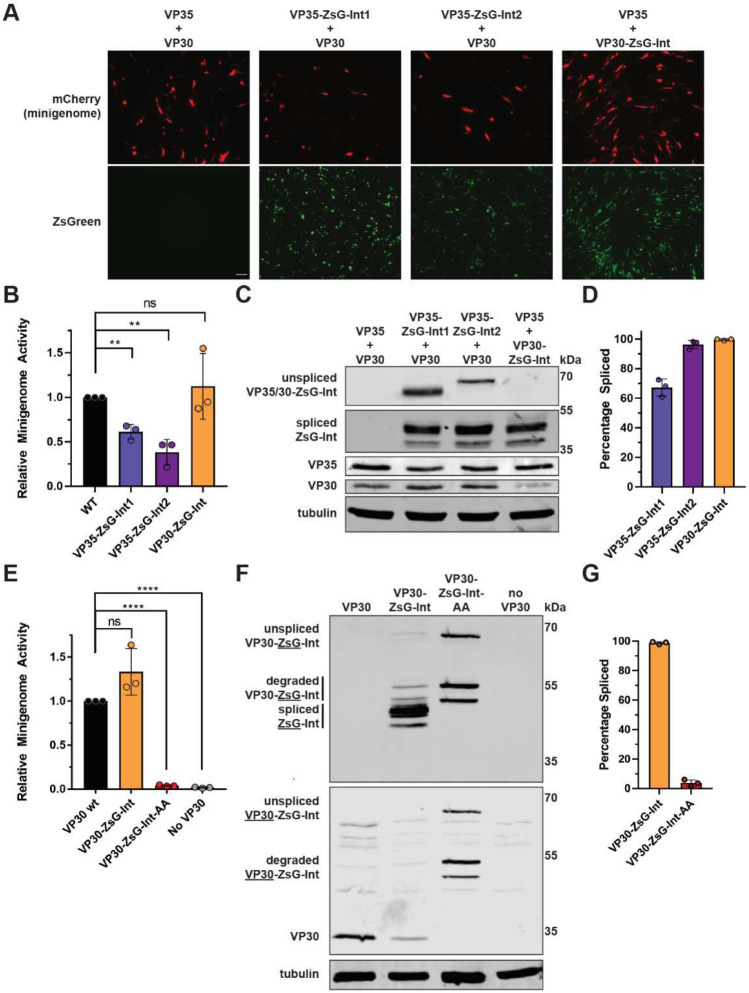
Functional assessment of VP35 and VP30 constructs containing ZsG-Int. (**A**) Minigenome activity (mCherry, red) of BHK21 cells expressing wild-type EBOV system components versus those substituted with the indicated ZsG-Int constructs. ZsG-Int expression (ZsGreen, green). Scale bar = 100 µm. Images were taken with 10× objective. (**B**) Minigenome activity (relative NanoLuc activity) of Huh7 cells expressing wild-type EBOV system components versus those substituted with the indicated ZsG-Int constructs. ** = *p* < 0.01, ns = not significant. (**C**) Western blot analysis of cell lysates from (**B**). (**D**) Protein splicing efficiencies from C based on 3 replicates were quantified and calculated as in Figure 2D. (**E**) Minigenome activity (relative NanoLuc activity) of Huh7 cells expressing wild-type EBOV VP30, VP30-ZsG-Int, VP30-ZsG-Int-AA, or no VP30. **** = *p* < 0.0001, ns = not significant. (**F**) Western blot analysis of cell lysates from (**E**). Numbers on the right indicate molecular mass of protein marker bands. Antibodies detecting ZsGreen and VP30 were used for the top and middle blots, respectively. (**G**) Protein splicing efficiency from (**G**) based on 3 replicates was quantified and calculated as in Figure 2D. Experiments have been performed 3 times with similar outcomes. Representative results are shown in (**A**,**C**,**F**).

We next tested the functionality of these intein-containing proteins using EBOV minigenome systems [26]. Minigenome systems are transfection-based reporter systems for assessing viral transcription and replication where a minigenome consisting of viral genomic termini flanking a reporter gene is co-expressed with the viral proteins required for viral genome replication and transcription. In the case of EBOV, these proteins, the so-called system components, include the nucleoprotein NP and the RNA-dependent RNA polymerase L in addition to VP35 and VP30 [31]. Replication and transcription of the minigenome by the system components can be monitored by reporter gene expression. We first used an EBOV minigenome expressing a mCherry reporter gene to determine the functionality of the ZsG-Int-containing VP35 and VP30 constructs. Expression of wild-type L, NP, VP35, and VP30 resulted in efficient minigenome activity as determined by mCherry fluorescence (Figure 3A). Replacing wild-type VP30 with VP30-ZsG-Int resulted in robust reporter activity, whereas the use of VP35-ZsG-Int1 or VP35-ZsG-Int2 in place of wild-type VP35 resulted in reduced minigenome activity (Figure 3A). Using an EBOV minigenome containing a NanoLuc reporter gene we similarly observed that using VP35-ZsG-Int1 or VP35-ZsG-Int2 instead of wild-type VP35 significantly reduced minigenome activity despite similar VP35 expression levels but that replacing wild-type VP30 with VP30-ZsG-Int did not alter minigenome activity despite lower VP30 expression (Figure 3B,C and Figure S2). The splicing efficiency of the ZsG-Int-containing proteins in minigenome transfected cells mirrored what was seen when they were individually expressed (Figure 2D vs. Figure 3D). However, the reduction in activity observed for VP35 fusions may be due to off-pathway reactions, where the N- or C-extein is irreversibly cleaved from the intein prior to ligation, as the size of the C-extein for VP35-ZsG-Int1 and N-extein for VP35-ZsG-Int2 are similar in size to the ligated exteins.

To assess the functionality of unspliced VP30-ZsG-Int, the N- and C-terminal residues of the intein (cysteine 263 and asparagine 671) were mutated to alanine (VP30-ZsG-Int-AA) which should render the intein incapable of splicing [25]. Replacing wt VP30 with VP30-ZsG-Int-AA resulted in a near complete loss of minigenome reporter activity (Figure 3E), despite efficient VP30-ZsG-Int-AA expression (Figure 3F). The greatly reduced minigenome activity is similar to what was observed when VP30 was omitted from the minigenome system (Figure 3E), as has been previously described for EBOV [26], showing that VP30-ZsG-Int-AA is nonfunctional and indicating that efficient splicing may be required for VP30-ZsG-Int function [31]. Western blot analysis showed that although VP30-ZsG-Int-AA was efficiently expressed, splicing activity was severely compromised as evidenced by the lack of appropriately sized VP30 or ZsG-Int (Figure 3F,G).

### 3.4. Generation and Analysis of Recombinant Ebola Virus Containing an Intein-Based Reporter

**Figure 4:**
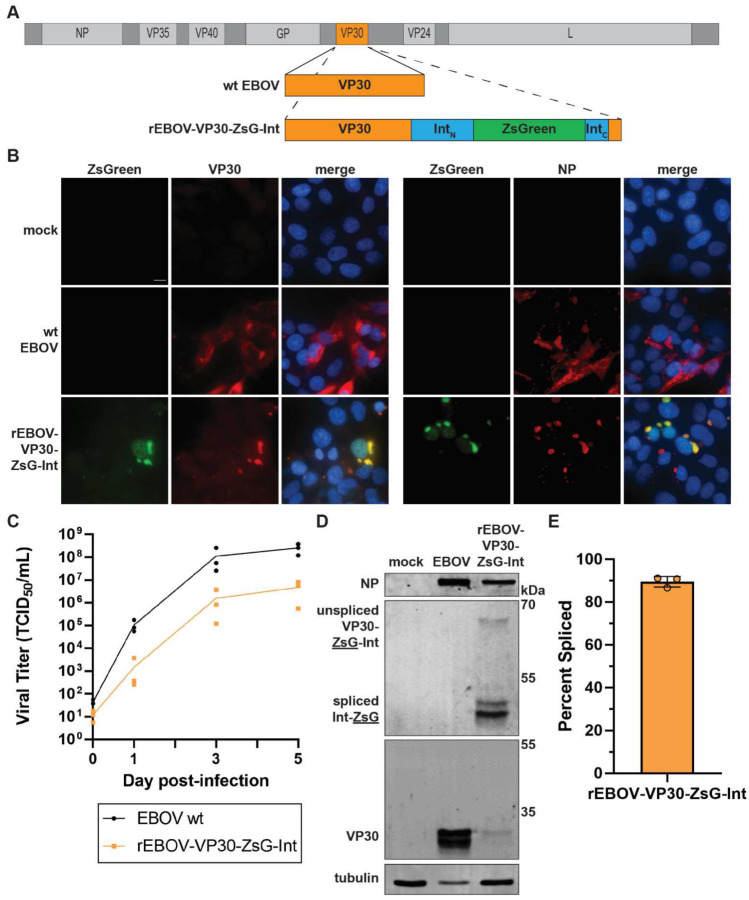
Rescue of recombinant EBOV containing VP30-ZsG-Int. (**A**) Diagram of wt EBOV genome versus rEBOV-VP30-ZsG-Int. Diagram is to scale. VP30 ORF in orange, other ORFs in light gray, intein N terminus (Int-N) and C terminus (Int-C) in blue, ZsGreen in green, non-coding regions in dark gray. (**B**) Fluorescence microscopy of Huh7 cells infected with wt EBOV or rEBOV-VP30-ZsG-Int at a multiplicity of infection (MOI) of 3. Cells were fixed at 1 day post-infection (dpi) and stained for EBOV VP30 or NP as indicated. Scale bar = 10 µm. Images were taken with 100× objective. (**C**) Growth curve comparing wt EBOV and rEBOV-VP30-ZsG-Int. Vero E6 cells were infected at an MOI of 0.1. (**D**) Western blot analysis of Huh7 cells infected with the indicated viruses at an MOI of 3. Cell lysates were harvested at 2 dpi. Molecular mass of protein marker bands is indicated on the right. (**E**) Splicing efficiency from D were quantified from 3 experiments and calculated as in Figure 2D. Experiments have been performed 3 times with similar outcomes, with representative results shown in (**B**,**D**).

Combined, these data indicated that the VP30-ZsG-Int construct is highly efficient at splicing, leaving VP30 fully functional. To analyze whether this intein-containing viral gene could effectively be used to generate a recombinant version of EBOV expressing a fluorescent reporter, we incorporated VP30-ZsG-Int into a full-length EBOV construct, replacing VP30 (Figure 4A). We were able to rescue the corresponding recombinant virus (rEBOV-VP30-ZsG-Int) by transfecting cells with this full-length construct along with the EBOV system components, as previously described [32]. Cells infected with rEBOV-VP30-ZsG-Int showed ZsGreen fluorescence which was primarily colocalized with viral inclusions, as evidenced by colocalization with both VP30 and NP, with small amounts localized to the nucleus as has sometimes been observed for ZsGreen [33] (Figure 4B and Figure S3). This fluorescence was maintained throughout the viral infection and was not lost during three serial passages of the virus. rEBOV-VP30-ZsG-Int was attenuated compared to wt EBOV (Figure 4C) despite efficient VP30-ZsG-Int splicing (Figure 4D,E). This was reflected by a lower infection rate as shown by immunofluorescence (Figure 4B and Figure S3), delayed production of viral proteins NP and VP30 as shown by Western blot analysis (Figure 4D), and slower viral replication kinetics (Figure 4C). The two bands of VP30 observed by Western blot in infected cells likely represent unphosphorylated and phosphorylated VP30 as has been seen previously [34] (Figure 4D). The low concentration of VP30 at early times in cells infected with rEBOV-VP30-ZsG-Int may contribute to the growth delay observed for this virus (Figure 4C,D). Despite these limitations, cells infected with rEBOV-VP30-ZsG-Int express bright ZsGreen fluorescence, and therefore, represents an effective initial attempt at labeling a virus with an intein-based reporter.

## 4. Discussion

Here, we describe a novel reporter gene expression system based on inteins that functions in both prokaryotic and eukaryotic cells, suggesting a broad application potential. The ZsG-Int reporter is unique in that it is only made if the protein of interest is translated, but it does not remain part of the final product. The use of modified inteins provides a novel technique for inserting reporter genes into viral genomes or other biological systems without the need to replace or create additional transcriptional units. While inserting a ZsG-Int reporter into the VP30 gene of EBOV was tolerated and led to reporter gene expression, the resulting recombinant virus was attenuated compared to an unlabeled virus, likely due to low VP30 expression potentially caused by suboptimal intein splicing at early stages of infection. Future work will aim to improve both the speed and efficiency of the reporter splicing and better define ideal ZsG-Int insertion sites to avoid a growth defect. These data nevertheless provide proof of principle, suggesting great potential for this approach of using modified inteins as self-excising translational reporters.

## Data Availability

Data is contained within the article or supplementary material.

## Supplementary Materials

The following supporting information can be downloaded at: https://www.mdpi.com/article/10.3390/microorganisms10112110/s1, Figure S1: Uncropped version of Western blots shown in Figure 2C; Figure S2: Uncropped version of Western blots shown in Figure 3C; Figure S3: Individual channels from Figure 4B; File S1: Sequences of ordered DNA fragments.
