# Supplementary material for: Labeling Ebola Virus with a Self-Splicing Fluorescent Reporter
Source: Microorganisms. 2022 Oct 26;10(11):2110. doi: 10.3390/microorganisms10112110 (PMC9696229; doi:10.3390/microorganisms10112110)
Supplement: Supplementary file 1 [file microorganisms-10-02110-s001.zip › microorganisms-1972351-supplementary.pdf]

File S1: Sequences of ordered DNA fragments.

Sequences used in the study

The ZsG-Int protein sequence is as follows:

CFARDTEVYYENDTVPHMESIEEMYSKYASMNGELPFDNGYAVPLDNVFVYTLDIASGEIKKTR
ASYIYREKVEKLIEIKLSSGYSLKVTPSHPVLLFRDGLQWVPAAEVKPGDVVVGVREEVLRRGS
GMAQSKHGLTKEMTMKYRMEGCVDGHKFVITGEGIGYPFKGKQAINLCVVEGGPLPFAEDILS
AAFMYGNRVFTEYPQDIVDYFKNSCPAGYTWDRSFLFEDGAVCICNADITVSVEENCMYHESK
FYGVNFPADGPVMKKMTDNWEPSCEKIIPVPKQGILKGDVSMYLLLKDGGRLRCQFDTVYKAK
SVPRKMPDWHFIQHKLTREDRSDAKNQKWHLTEHAIASGSALPGSGRIISKGELEFHEVSSVRII
DYNNWVYDLVIPETHNFIAPNGLVLHN

Intein residues are in red, linker residues are in black, and ZsGreen residues are in green. The ZsG-Int residues were flanked by the Maltose Binding Protein (*E. coli malE* residues 27-392) followed by residues GSGK (N-extein) and TQLAHTLAVMVHHHHHH (C-extein).

A pTwist-Amp plasmid containing the ZsG-Int1 insert for VP35 was ordered from Twist Bioscience as follows:

GACGTCTCATCATTTTGGCAAAGAATTCCTCGAGGGTACCCCCGGGGAGCTCACTAGTGG
ATCCAAACCTTCATCATCCTTACGTCAATTGAATTCTCTAGCACTCGAAGCTTATTGTCTTCA
ATGTAAAAGAAAAGCTGGTCTAACAAGATGACAACTAGAACAAAGGGCAGGGGCCATTGCT
TCGCTAGAGACACCGAGGTGTACTACGAGAACGACACCGTGCCCCACATGGAGAGCATCG
AGGAGATGTACAGCAAGTACGCTAGCATGAACGGCGAGCTGCCCTTCGACAACGGCTACG
CCGTGCCCCTGGACAACGTGTTCGTGTACACCCTGGACATCGCTAGCGGCGAGATCAAGA
AGACAAGAGCTAGCTACATCTACAGAGAGAAGGTGGAGAAGCTGATCGAGATCAAGCTGA
GCAGCGGCTACAGCCTGAAGGTGACCCCTAGCCACCCGGTGCTGCTGTTCAGAGATGGC
CTGCAGTGGGTGCCCGCGGCCGAGGTGAAGCCCGGCGACGTGGTGGTGGGCGTGAGAG
AAGAGGTGCTGAGAAGAGGCAGCGGCATGGCCCAGTCCAAGCACGGCCTGACCAAGGAG
ATGACCATGAAGTACCGCATGGAGGGCTGCGTGGACGGCCACAAGTTCGTGATCACCGG
CGAGGGCATCGGCTACCCCTTCAAGGGCAAGCAGGCCATCAACCTGTGCGTGGTGGAGG
GCGGCCCCTTGCCCTTCGCCGAGGACATCTTGTCCGCCGCCTTCATGTACGGCAACCGCG
TGTTCACCGAGTACCCCCAGGACATCGTCGACTACTTCAAGAACTCCTGCCCCGCCGGCT
ACACCTGGGACCGCTCCTTCCTGTTCGAGGACGGCGCCGTGTGCATCTGCAACGCCGACA
TCACCGTGAGCGTGGAGGAGAACTGCATGTACCACGAGTCCAAGTTCTACGGCGTGAACT
TCCCCGCCGACGGCCCCGTGATGAAGAAGATGACCGACAACTGGGAGCCCTCCTGCGAG
AAGATCATCCCCGTGCCCAAGCAGGGCATCTTGAAGGGCGACGTGAGCATGTACCTGCTG
CTGAAGGACGGTGGCCGCTTGCGCTGCCAGTTCGACACCGTGTACAAGGCCAAGTCCGT
GCCCCGCAAGATGCCCGACTGGCACTTCATCCAGCACAAGCTGACCCGCGAGGACCGCA
GCGACGCCAAGAACCAGAAGTGGCACCTGACCGAGCACGCCATCGCCTCCGGCTCCGCC
TTGCCCGGAAGCGGCAGAATCATCAGCAAGGGCGAGCTGGAGTTCCACGAGGTGAGCAG
CGTGAGAATCATCGACTACAACAACTGGGTGTACGACCTGGTGATCCCCGAGACCCACAA
CTTCATCGCCCCCAACGGCCTGGTGCTGCACAACACTGCGGCCACGACTCAAAACGACAG
AATGCCAGGCCCTGAGCTTTCGGGCTGGATCTCTGAGCAGCTAATGACCGGAAGAATTCC
TGTAAGCGACATCTTCTGTGATATTGAGAACAATCCAGGATTATGCTACGCATCCCAAATGC
AACAAACGAAGCCAAACCCGAAGACGCGCAACAGTCAAACCCAAACGGACCCAATTTGCA
ATCATAGTTTTGAGGAGGTAGTACAAACATTGGCTTCATTGGCTACTGTTGTGCAACAACAA
ACCATCGCATCAGAATCATTAGAACAACGCATTACGAGTCTTGAGAATGGTCTAAAGCCAG
TTTATGATATGGCAAAAACAATCTCCTCATTGAACAGGGTTTGTGCTGAGATGGTTGCAAAA

TATGATCTTCTGGTGATGACAACCGGTCGGGCAACAGCAACCGCTGCGGCAACTGAGGCT
TATTGGGCCGAACATGGTCAACCACCACCTGGACCATCACTTTATGAAGAAAGTACT

EBOV VP35 sequence in blue, intein sequence is in red, linker sequences are in black, and ZsGreen sequence is in green. pCAGGS-VP35$_{EBOV}$ was digested with KpnI, AgeI, and DpnI and the Genscript vector was digested with ZraI and ScaI-HF (NEB). Fragments were gel purified and cloned using NEBuilder HiFi DNA Assembly (NEB).

A pTwist-Amp plasmid containing the ZsG-Int2 insert for VP35 was ordered from Twist Bioscience as follows:

TTTAAAATATGATCTTCTGGTGATGACAACCGGTCGGGCAACAGCAACCGCTGCGGCAACT
GAGGCTTATTGGGCCGAACATGGTCAACCACCACCTGGACCATCACTTTATGAAGAAAGTG
CGATTCGGGGTAAGATTGAATCTAGAGATGAGACCGTCCCTCAAAGTGTTAGGGAGGCATT
CAACAATCTAAACAGTACCACTTCACTAACTGAGGAAAATTTTGGGAAACCTGACATTTCGG
CAAAGGATTTGAGAAACATTATGTATGATCACTTGCCTGGTTTTGGAACTGCTTTCCACCAA
TTAGTACAAGTGATTTGTAAATTGGGAAAAGATAGCAACTCATTGGACATCATTCATGCTGA
GTTCCAGGCCAGCCTGGCTGAAGGAGACTCTCCTCAATGTGCCCTAATTCAAATTACAAAA
AGAGTTCCAATCTTCCAAGATGCTGCTCCACCTGTCATCCACATCCGCTCTCGAGGTGACA
TTCCCCGAGCTTGCCAGAAAAGCTTGCGTCCAGTCCCACCATCGCCCAAGATTGATCGAG
GTTGGGTATGTGTTTTTCAGCTTCAAGATGGTAAATGCTTCGCTAGAGACACCGAGGTGTA
CTACGAGAACGACACCGTGCCCCACATGGAGAGCATCGAGGAGATGTACAGCAAGTACGC
TAGCATGAACGGCGAGCTGCCCTTCGACAACGGCTACGCCGTGCCCCTGGACAACGTGTT
CGTGTACACCCTGGACATCGCTAGCGGCGAGATCAAGAAGACAAGAGCTAGCTACATCTA
CAGAGAGAAGGTGGAGAAGCTGATCGAGATCAAGCTGAGCAGCGGCTACAGCCTGAAGG
TGACCCCTAGCCACCCGGTGCTGCTGTTCAGAGATGGCCTGCAGTGGGTGCCCGCGGCC
GAGGTGAAGCCCGGCGACGTGGTGGTGGGCGTGAGAGAAGAGGTGCTGAGAAGAGGCA
GCGGCATGGCCCAGTCCAAGCACGGCCTGACCAAGGAGATGACCATGAAGTACCGCATG
GAGGGCTGCGTGGACGGCCACAAGTTCGTGATCACCGGCGAGGGCATCGGCTACCCCTT
CAAGGGCAAGCAGGCCATCAACCTGTGCGTGGTGGAGGGCGGCCCCTTGCCCTTCGCCG
AGGACATCTTGTCCGCCGCCTTCATGTACGGCAACCGCGTGTTCACCGAGTACCCCCAGG
ACATCGTCGACTACTTCAAGAACTCCTGCCCCGCCGGCTACACCTGGGACCGCTCCTTCC
TGTTCGAGGACGGCGCCGTGTGCATCTGCAACGCCGACATCACCGTGAGCGTGGAGGAG
AACTGCATGTACCACGAGTCCAAGTTCTACGGCGTGAACTTCCCCGCCGACGGCCCCGTG
ATGAAGAAGATGACCGACAACTGGGAGCCCTCCTGCGAGAAGATCATCCCCGTGCCCAAG
CAGGGCATCTTGAAGGGCGACGTGAGCATGTACCTGCTGCTGAAGGACGGTGGCCGCTT
GCGCTGCCAGTTCGACACCGTGTACAAGGCCAAGTCCGTGCCCCGCAAGATGCCCGACT
GGCACTTCATCCAGCACAAGCTGACCCGCGAGGACCGCAGCGACGCCAAGAACCAGAAG
TGGCACCTGACCGAGCACGCCATCGCCTCCGGCTCCGCCTTGCCCGGAAGCGGCAGAAT
CATCAGCAAGGGCGAGCTGGAGTTCCACGAGGTGAGCAGCGTGAGAATCATCGACTACAA
CAACTGGGTGTACGACCTGGTGATCCCCGAGACCCACAACTTCATCGCCCCCAACGGCCT
GGTGCTGCACAACACACTTGGACTCAAAATTTGAGCCAATCTCCCTTCCCTCCGAAAGAGG
CGAATAATAGCAGAGGCTTCAACTGCTGAACTATAGGGTACGTTACATTAATGATACACTTG
TGAGTATCAGCCCTGGATAATATAAGTCAATTAAACGACCAAGATAAAATTGTTCATATCTC
GCTAGCAGATCTTTTTCCCTCTGCCAAAAATTATAA

EBOV VP35 sequence in blue, intein sequence is in red, linker sequences are in black, and ZsGreen sequence is in green. pCAGGS-VP35$_{EBOV}$ was digested with AgeI, NheI, and DpnI and the Genscript vector was digested with DraI and PsiI-v2 (NEB). Fragments were gel purified and cloned using NEBuilder HiFi DNA Assembly (NEB).

A pTwist-Amp plasmid containing the ZsG-Int insert for VP30 (pTwist-Amp-ZsG-Int) was ordered from Twist Bioscience as follows:

CAGCTGAGTCTTTTATGTGAGACACACCTAAGGCGCGAGGGGCTTGGGCAAGATCAGGCA
GAACCCGTTCTCGAAGTATATCAACGATTACACAGTGATAAAGGAGGCAGTTTTGAAGCTG
CACTATGGCAACAATGGGACCGACAATCCCTAATTATGTTTATCACTGCATTCTTGAATATT
GCTCTCCAGTTACCGTGTGAAAGTTCTGCTGTCGTTGTTTCAGGGTTAAGATGCTTCGCTA
GAGACACCGAGGTGTACTACGAGAACGACACCGTGCCCCACATGGAGAGCATCGAGGAG
ATGTACAGCAAGTACGCTAGCATGAACGGCGAGCTGCCCTTCGACAACGGCTACGCCGTG
CCCCTGGACAACGTGTTCGTGTACACCCTGGACATCGCTAGCGGCGAGATCAAGAAGACA
AGAGCTAGCTACATCTACAGAGAGAAGGTGGAGAAGCTGATCGAGATCAAGCTGAGCAGC
GGCTACAGCCTGAAGGTGACCCCTAGCCACCCGGTGCTGCTGTTCAGAGATGGCCTGCA
GTGGGTGCCCGCGGCCGAGGTGAAGCCCGGCGACGTGGTGGTGGGCGTGAGAGAAGAG
GTGCTGAGAAGAGGCAGCGGCATGGCCCAGTCCAAGCACGGCCTGACCAAGGAGATGAC
CATGAAGTACCGCATGGAGGGCTGCGTGGACGGCCACAAGTTCGTGATCACCGGCGAGG
GCATCGGCTACCCCTTCAAGGGCAAGCAGGCCATCAACCTGTGCGTGGTGGAGGGCGGC
CCCTTGCCCTTCGCCGAGGACATCTTGTCCGCCGCCTTCATGTACGGCAACCGCGTGTTC
ACCGAGTACCCCCAGGACATCGTCGACTACTTCAAGAACTCCTGCCCCGCCGGCTACACC
TGGGACCGCTCCTTCCTGTTCGAGGACGGCGCCGTGTGCATCTGCAACGCCGACATCACC
GTGAGCGTGGAGGAGAACTGCATGTACCACGAGTCCAAGTTCTACGGCGTGAACTTCCCC
GCCGACGGCCCCGTGATGAAGAAGATGACCGACAACTGGGAGCCCTCCTGCGAGAAGAT
CATCCCCGTGCCCAAGCAGGGCATCTTGAAGGGCGACGTGAGCATGTACCTGCTGCTGAA
GGACGGTGGCCGCTTGCGCTGCCAGTTCGACACCGTGTACAAGGCCAAGTCCGTGCCCC
GCAAGATGCCCGACTGGCACTTCATCCAGCACAAGCTGACCCGCGAGGACCGCAGCGAC
GCCAAGAACCAGAAGTGGCACCTGACCGAGCACGCCATCGCCTCCGGCTCCGCCTTGCC
CGGAAGCGGCAGAATCATCAGCAAGGGCGAGCTGGAGTTCCACGAGGTGAGCAGCGTGA
GAATCATCGACTACAACAACTGGGTGTACGACCTGGTGATCCCCGAGACCCACAACTTCAT
CGCCCCCAACGGCCTGGTGCTGCACAACACATTGGTTCCTCAATCAGATAATGAGGAAGC
TTCAACCAACCCGGGGACATGCTCATGGTCTGATGAGGGTACCCCTTAATAAGGCTGACTA
AAACACTATATAACCTTCTACTTGATCACAATACTCCGTATACCTATCATCATATATTTAATC
AAGACGATATC

EBOV VP30 sequence in blue, intein sequence is in red, linker sequences are in black, and ZsGreen sequence is in green. pCAGGS-VP30$_{EBOV}$ was digested with Bsu36I, BstZ171-HF and DpnI and the Genscript vector was digested with PvuII-HF and EcorRV (NEB). Fragments were gel purified and cloned using NEBuilder HiFi DNA Assembly (NEB).

A DNA fragment containing the ZsG-Int-AA insert for VP30 was ordered from Twist Bioscience as follows:

GACGAGGAAATTCTCAAAATCCCAGCTGAGTCTTTTATGTGAGACACACCTAAGGC
GCGAGGGGCTTGGGCAAGATCAGGCAGAACCCGTTCTCGAAGTATATCAACGATT
ACACAGTGATAAAGGAGGCAGTTTTGAAGCTGCACTATGGCAACAATGGGACCGA
CAATCCCTAATTATGTTTATCACTGCATTCTTGAATATTGCTCTCCAGTTACCGTGT
GAAAGTTCTGCTGTCGTTGTTTCAGGGTTAAGAGCGTTCGCTAGAGACACCGAGGT
GTACTACGAGAACGACACCGTGCCCCACATGGAGAGCATCGAGGAGATGTACAGC
AAGTACGCTAGCATGAACGGCGAGCTGCCCTTCGACAACGGCTACGCCGTGCCC
CTGGACAACGTGTTCGTGTACACCCTGGACATCGCTAGCGGCGAGATCAAGAAGA

CAAGAGCTAGCTACATCTACAGAGAGAAGGTGGAGAAGCTGATCGAGATCAAGCT
GAGCAGCGGCTACAGCCTGAAGGTGACCCCTAGCCACCCGGTGCTGCTGTTCAG
AGATGGCCTGCAGTGGGTGCCCGCGGCCGAGGTGAAGCCCGGCGACGTGGTGG
TGGGCGTGAGAGAAGAGGTGCTGAGAAGAGGCAGCGGCATGGCCCAGTCCAAGC
ACGGCCTGACCAAGGAGATGACCATGAAGTACCGCATGGAGGGCTGCGTGGACG
GCCACAAGTTCGTGATCACCGGCGAGGGCATCGGCTACCCCTTCAAGGGCAAGC
AGGCCATCAACCTGTGCGTGGTGGAGGGCGGCCCCTTGCCCTTCGCCGAGGACA
TCTTGTCCGCCGCCTTCATGTACGGCAACCGCGTGTTCACCGAGTACCCCCAGGA
CATCGTCGACTACTTCAAGAACTCCTGCCCCGCCGGCTACACCTGGGACCGCTCC
TTCCTGTTCGAGGACGGCGCCGTGTGCATCTGCAACGCCGACATCACCGTGAGCG
TGGAGGAGAACTGCATGTACCACGAGTCCAAGTTCTACGGCGTGAACTTCCCCGC
CGACGGCCCCGTGATGAAGAAGATGACCGACAACTGGGAGCCCTCCTGCGAGAA
GATCATCCCCGTGCCCAAGCAGGGCATCTTGAAGGGCGACGTGAGCATGTACCTG
CTGCTGAAGGACGGTGGCCGCTTGCGCTGCCAGTTCGACACCGTGTACAAGGCC
AAGTCCGTGCCCCGCAAGATGCCCGACTGGCACTTCATCCAGCACAAGCTGACCC
GCGAGGACCGCAGCGACGCCAAGAACCAGAAGTGGCACCTGACCGAGCACGCCA
TCGCCTCCGGCTCCGCCTTGCCCGGAAGCGGCAGAATCATCAGCAAGGGCGAGC
TGGAGTTCCACGAGGTGAGCAGCGTGAGAATCATCGACTACAACAACTGGGTGTA
CGACCTGGTGATCCCCGAGACCCACAACTTCATCGCCCCCAACGGCCTGGTGCTG
CACGCCACATTGGTTCCTCAATCAGATAATGAGGAAGCTTCAACCAACCCGGGGA
CATGCTCATGGTCTGATGAGGGTACCCCTTAATAAGGCTGACTAAAACACTATATA
ACCTTCTACTTGATCACAATACTCCGTATACCTATCATCATATATTTAATCAAGACGA
TATCCTTTAAAACTTATTCAGTACTA

EBOV VP30 sequence in blue, intein sequence is in red, linker sequences are in black, and ZsGreen sequence is in green. pCAGGS-VP30$_{EBOV}$ was digested with PvuII-HG, EcoRV, and DpnI and gel purified. Fragments were cloned using NEBuilder HiFi DNA Assembly (NEB).

Supplemental Figures

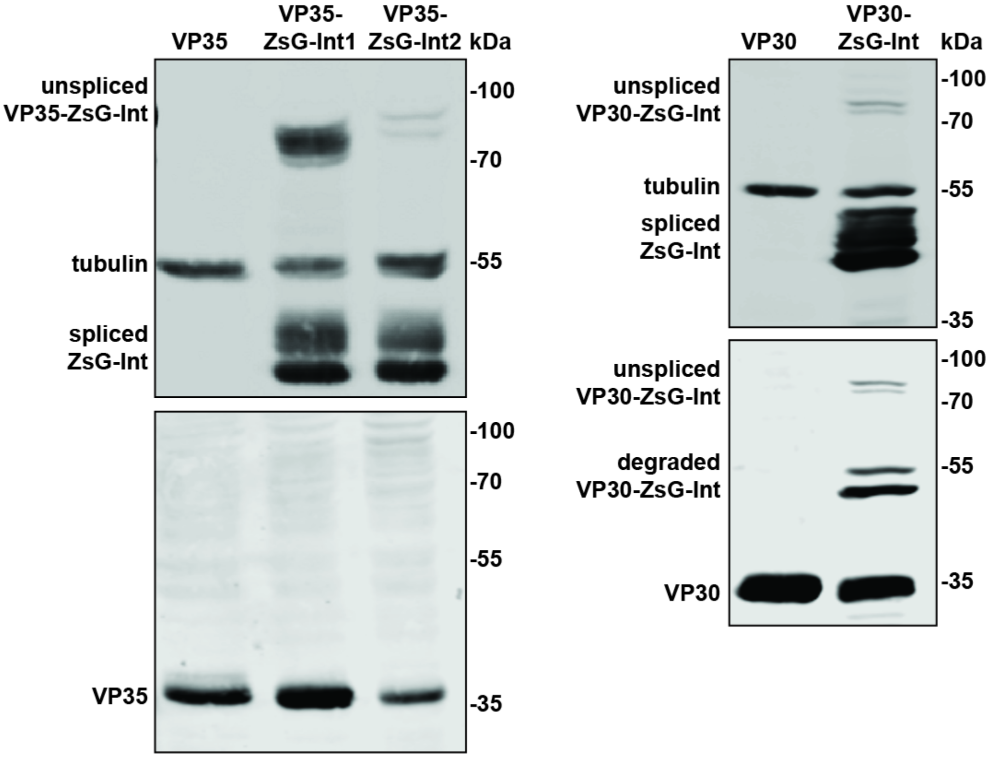

Figure S1. Uncropped version of Western blots shown in Figure 2C. Western blots of 293T cells expressing wt and ZsG-Int-containing VP35 and VP30 constructs at 2 dpt. Detection of unspliced proteins VP35-ZsG-Int1/2 (83.6 kDa) and VP30-ZsG-Int (78.8 kDa) as well as spliced products ZsG-Int (46.2 kDa), VP35 (37.4 kDa), and VP30 (32.6 kDa).

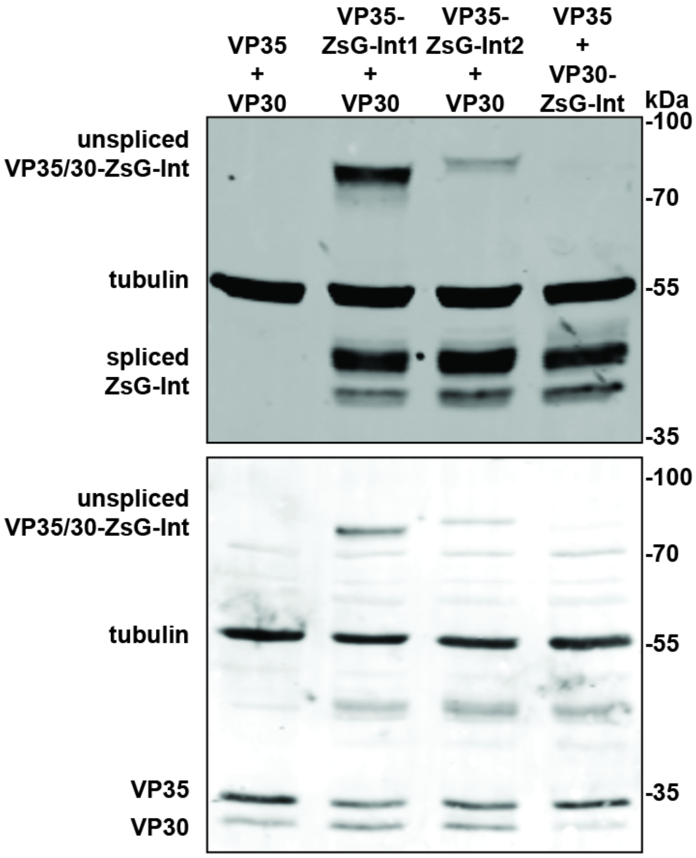

Figure S2. Uncropped version of Western blots shown in Figure 3C. Western blot analysis of Huh7 cell lysates from minigenome transfected cells expressing wild-type EBOV system components versus those substituted with the indicated ZsG-Int constructs.

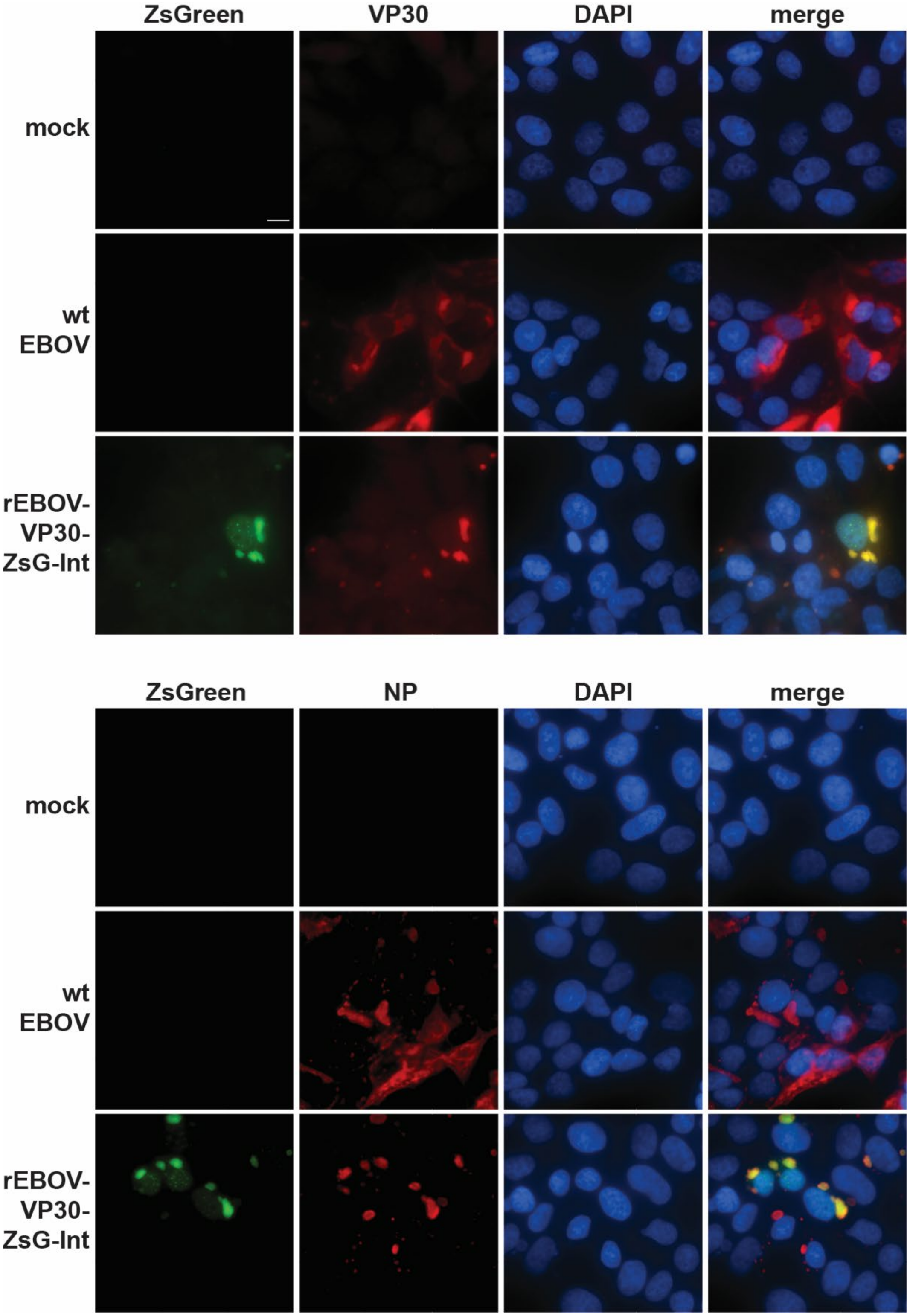

Figure S3. Individual channels from Figure 4B. Fluorescence microscopy of Huh7 cells infected with wt EBOV or rEBOV-VP30-ZsG-Int at a multiplicity of infection (MOI) of 3. Cells were fixed at 1 day post infection (dpi) and stained for EBOV VP30 or NP as indicated. Scale bar = 10 µM. Images were taken with 100x objective.
